# Transcriptomic Study of Substrate-Specific Transport Mechanisms for Iron and Carbon in the Marine Copiotroph Alteromonas macleodii

**DOI:** 10.1128/mSystems.00070-20

**Published:** 2020-04-28

**Authors:** Lauren E. Manck, Josh L. Espinoza, Christopher L. Dupont, Katherine A. Barbeau

**Affiliations:** a Geosciences Research Division, Scripps Institution of Oceanography, University of California San Diego, La Jolla, California, USA; b Department of Environment and Sustainability, J. Craig Venter Institute, La Jolla, California, USA; c Department of Human Health, J. Craig Venter Institute, La Jolla, California, USA; d Department of Synthetic Biology, J. Craig Venter Institute, La Jolla, California, USA; University of Hawaii at Manoa

**Keywords:** *Alteromonas*, TonB, carbon transport, gene expression, iron transport, marine microbiology

## Abstract

Iron is an essential micronutrient for all microbial growth in the marine environment, and in heterotrophic bacteria, iron is tightly linked to carbon metabolism due to its central role as a cofactor in enzymes of the respiratory chain. Here, we present the iron- and carbon-regulated transcriptomes of a representative marine copiotroph, Alteromonas macleodii ATCC 27126, and characterize its cellular transport mechanisms. ATCC 27126 has distinct metabolic responses to iron and carbon limitation and, accordingly, uses distinct sets of TonB-dependent transporters for the acquisition of iron and carbon. These distinct sets of TonB-dependent transporters were of a similar number, indicating that the diversity of carbon and iron substrates available to ATCC 27126 is of a similar scale. For the first time in a marine bacterium, we have also identified six characteristic inner membrane permeases for the transport of siderophores via an ATPase-independent mechanism. An examination of the distribution of specific TonB-dependent transporters in 31 genomes across the genus Alteromonas points to niche specialization in transport capacity, particularly for iron. We conclude that the substrate-specific bioavailability of both iron and carbon in the marine environment will likely be a key control on the processing of organic matter through the microbial loop.

**IMPORTANCE** As the major facilitators of the turnover of organic matter in the marine environment, the ability of heterotrophic bacteria to acquire specific compounds within the diverse range of dissolved organic matter will affect the regeneration of essential nutrients such as iron and carbon. TonB-dependent transporters are a prevalent cellular tool in Gram-negative bacteria that allow a relatively high-molecular-weight fraction of organic matter to be directly accessed. However, these transporters are not well characterized in marine bacteria, limiting our understanding of the flow of specific substrates through the marine microbial loop. Here, we characterize the TonB-dependent transporters responsible for iron and carbon acquisition in a representative marine copiotroph and examine their distribution across the genus *Alteromonas*. We provide evidence that substrate-specific bioavailability is niche specific, particularly for iron complexes, indicating that transport capacity may serve as a significant control on microbial community dynamics and the resultant cycling of organic matter.

## Abstract (precis)

As the major facilitators of the turnover of organic matter in the marine environment, the ability of heterotrophic bacteria to acquire specific compounds within the diverse range of dissolved organic matter will affect the regeneration of essential nutrients such as iron and carbon. TonB-dependent transporters are a prevalent cellular tool in Gram-negative bacteria that allow a relatively high-molecular-weight fraction of organic matter to be directly accessed. However, these transporters are not well characterized in marine bacteria, limiting our understanding of the flow of specific substrates through the marine microbial loop. Here, we characterize the TonB-dependent transporters responsible for iron and carbon acquisition in a representative marine copiotroph and examine their distribution across the genus *Alteromonas*. We provide evidence that substrate-specific bioavailability is niche specific, particularly for iron complexes, indicating that transport capacity may serve as a significant control on microbial community dynamics and the resultant cycling of organic matter.

## INTRODUCTION

Iron (Fe) is an essential cofactor in many enzymes facilitating fundamental life processes, such as photosynthesis, respiration, and nitrogen fixation. As such, dissolved iron is a necessary micronutrient for all microbial growth in the marine environment and is tightly linked to the cycling of carbon (C) and other macronutrients. In oxygenated seawater, iron is most thermodynamically stable in the form of Fe(III) oxyhydroxides, which are characterized by low solubility and the tendency to be further scavenged by sinking particulate matter (1). This results in extremely low dissolved iron concentrations in most regions of the world’s oceans and exerts significant control on marine primary production (2).

Of the dissolved iron present in seawater, over 99% is associated with a heterogeneous pool of organic matter referred to as ligands (3). While much remains to be learned about the chemical composition of these ligands, they are thought to include humic substances, polysaccharides, metalloproteins with associated cofactors, and siderophores (4–8). Moreover, these ligands comprise a fraction of the total pool of marine dissolved organic matter (DOM). Marine DOM is one of the largest pools of carbon on Earth, and it is now recognized that marine heterotopic bacteria are the key determinant in the fate of this carbon and its associated macro- and micronutrients (9–11).

Given that marine DOM consists of a highly diverse matrix of organic compounds (12, 13), marine bacteria must use an assortment of cellular tools in order to access it. Many of the molecular mechanisms underlying these acquisition processes have yet to be explored. In particular, little is understood regarding these mechanisms at a molecular level for micronutrients such as iron. Measurements indicate that marine heterotrophic bacteria have iron quotas similar to or possibly greater than those of marine phytoplankton (14), and most of this iron resides within the respiratory chain, indicating a significant linkage between iron availability and carbon metabolism. Indeed, iron and carbon colimitation of marine heterotrophic communities has been observed (15). The specific uptake systems and enzymatic pathways that bacteria use to metabolize available substrates for both iron and carbon will influence rates of nutrient regeneration, the chemical composition of the remaining DOM, and, ultimately, the fate of fixed carbon within the ocean (12, 13, 16).

One identified pathway for the acquisition of organic complexes by prokaryotes is the use of TonB-dependent transporters (TBDTs). TBDTs transport larger compounds (generally greater than 600 Da) across the outer cell membrane in Gram-negative bacteria. The TBDT is coupled to the energizing proton motive force of the inner membrane through the TonB complex (TonB, ExbB, and ExbD). Subsequent transport across the inner membrane is often accomplished via an associated ATP-binding cassette transporter (ABCT). ABCTs also function independently of TBDTs for the transport of small substrates, such as inorganic Fe(III) or monomeric carbon substrates, that can diffuse across the outer membrane. Known compounds transported via TBDTs include those important to iron metabolism, such as Fe-siderophore complexes and heme, as well as solutes critical to carbon metabolism and cell growth, including amino acids, vitamins, and polysaccharides (17). Bioinformatic analysis of marine prokaryotic genomes and metagenomes reveals that TBDTs are fairly widespread and especially enriched in *Gammaproteobacteria* (18–20). However, sequence analysis alone cannot predict the substrate specificity of TBDTs, and very little is known about their regulation and use in marine heterotrophic bacteria.

Members of the genus Alteromonas are widespread marine copiotrophs of the class *Gammaproteobacteria*. The genus is globally distributed in oceanic waters, with several strains present in up to 80% of published samples from the *Tara* Oceans expedition (21). In addition, *Alteromonas* spp. have been found to become highly abundant in environments enriched in organic matter and nutrients (22–26). Observations of Southern Ocean bacterial communities have also found *Alteromonadaceae* to contribute significantly to the pool of iron uptake transcripts in this system (27). As “first responders” to bloom events and other episodes of particle enrichment, *Alteromonas* spp. play an important role in the biogeochemical cycling of organic matter (28). Due to their ability to disproportionately affect the processing of organic matter, we hypothesize that specific taxa will play a significant role in both carbon and iron remineralization processes.

We used Alteromonas macleodii ATCC 27126 (29) as an ecologically significant model bacterium in order to study the transport mechanisms underlying substrate-specific bioavailability of carbon and iron in the marine environment. We present iron- and carbon-regulated transcriptomes of *A. macleodii* ATCC 27126, with a focus on the expression of TBDTs and the regulation of central metabolic processes. This is the first study to directly compare the cellular response of a heterotrophic marine bacterium to both iron and carbon limitation under controlled conditions and reveals an unexpected contrast in the stress response to these two nutrient limitations. Additionally, we identified two distinct sets of TBDTs utilized for the transport of carbon and iron compounds and allowed for the putative identification of a wide range of specific substrates. These results give new insight into our understanding of substrate specific bioavailability of iron and carbon to the marine heterotrophic community and the potential effects this has on the biogeochemical cycling of organic matter in the marine environment.

## RESULTS

### Overview of global transcriptomic response to Fe and C limitation.

The differential expression of transcripts under iron and carbon limitation compared to that under iron- and carbon-replete conditions in *A. macleodii* ATCC 27126 was analyzed. Cultures for each nutrient condition were grown in biological triplicate (*n *= 3 for each of four treatments) in minimal medium tightly controlled for trace metal concentration and utilizing glucose as the carbon source (see Materials and Methods for details). While carbon-replete and iron-replete cultures are analyzed and discussed separately within the text, they represent two sets of biological triplicates grown under the same conditions. Limitation was defined as an observed decrease in growth in the absence of either added glucose or FeCl_3_ as monitored through optical density at 600 nm (OD_600_) measurements (see Fig. S1 in the supplemental material). While the removal of glucose did not result in the complete exclusion of carbon from the medium, the observed decrease in growth indicates that ATCC 27126 was carbon limited to a significant degree. Multiple iron and glucose concentrations were tested to achieve maximum growth under iron- and glucose-replete conditions (data not shown). The samples can be considered deeply sequenced, with 20 million to 30 million reads generated per sample, and transcripts from 3,892 of the 3,894 protein-coding genes (99.95%) within the *A. macleodii* ATCC 27126 genome were detected across all treatments and replicates. Transcripts for the remaining 2 protein-coding genes (MASE_02825 and MASE_13640) were absent from only one replicate each.

10.1128/mSystems.00070-20.1 FIG S1 Representative growth curves of Alteromonas macleodii ATCC 27126 under iron-depleted, carbon-depleted, and nutrient-replete conditions. Dashed lines indicate the growth state upon time of sampling. Download FIG S1, PDF file, 0.2 MB. Copyright © 2020 Manck et al. 2020 Manck et al. This content is distributed under the terms of the Creative Commons Attribution 4.0 International license.

**FIG 1:**
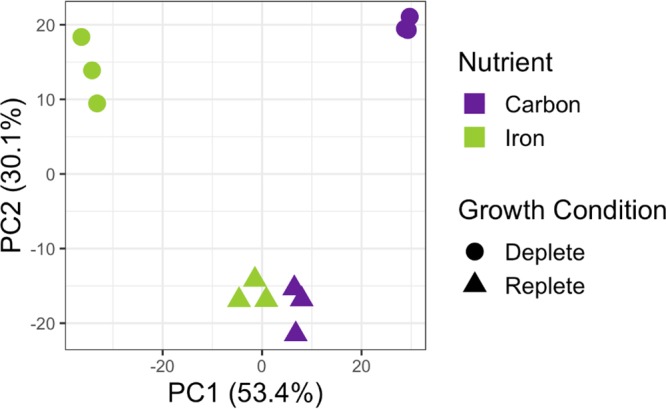
Ordination plot displaying the principal-component analysis of the normalized global transcript abundances of detected open reading frames of the Alteromonas macleodii ATCC 27126 genome under iron-depleted (green circles, *n *= 3), carbon-depleted (purple circles, *n *= 3), and nutrient-replete (green triangles, *n *= 3; and purple triangles, *n *= 3) conditions.

Principal-component (PC) analysis of the open reading frame (ORF) transcript abundances detected under each growth condition shows clear separation in global transcript expression patterns between iron-limited and carbon-limited states in ATCC 27126, while the six iron- and carbon-replete cultures cluster together (Fig. 1). The first two dimensions of the principal-component analysis account for 83.5% of the total variance, with 53.4% accounted for by the first dimension alone. Samples along PC1 are separated following a spectrum from iron depleted to carbon depleted, with the replete samples falling in the middle of this gradient. This reflects the unique transcriptional responses of ATCC 27126 to either iron or carbon limitation. In contrast, PC2 separates samples as strictly nutrient replete compared to nutrient depleted. Biological triplicates for each growth condition also cluster tightly together, yielding results with strong statistical significance. All fold change values are expressed as a comparison of transcript abundances under nutrient-limited versus nutrient-replete conditions. Using a fold change of ≥|2| and false-discovery rate (FDR) of <0.05 as thresholds for significance, we found that transcripts for 1,471 ORFs (38% of the total protein-coding genes) showed differential expression under at least one growth condition. Transcripts for 350 ORFs were significantly enriched (fold change, ≥2; FDR, <0.05) exclusively under iron-limited conditions, and those for 450 ORFs were uniquely enriched under carbon limitation, while only 78 ORFs were shared. Conversely, transcripts for 232 ORFs were uniquely depleted (fold change, ≤−2; FDR, <0.05) under iron limitation, while 358 ORFs were uniquely depleted under carbon limitation, and only 103 ORFs were shared. Note that transcripts enriched under iron limitation but depleted under carbon limitation (and vice versa) are not mutually exclusive. Specific ORFs discussed throughout the text are identified by gene locus (in the form MASE_XXXXX). See Data Set S1 for a complete list of differentially expressed ORFs and Fig. S2 for differential expression across the genome under both carbon and iron limitation.

10.1128/mSystems.00070-20.2 FIG S2 Differential expression of protein-coding genes along the Alteromonas macleodii ATCC 27126 genome under iron-depleted (green) and carbon-depleted (purple) conditions. Log_2_ fold change values are calculated with respect to nutrient-depleted conditions (i.e., positive values indicate enrichment under corresponding nutrient limitation). Shaded regions indicate log_2_ fold change values of <1 and >−1, which were not considered significant for subsequent analysis. See Data Set S1 for a complete list of differentially expressed ORFs (log_2_ fold change, ≥|1|; FDR, <0.05). Download FIG S2, PDF file, 0.3 MB. Copyright © 2020 Manck et al. 2020 Manck et al. This content is distributed under the terms of the Creative Commons Attribution 4.0 International license.

10.1128/mSystems.00070-20.8 DATA SET S1 Complete list of differentially expressed transcripts (log_2_ fold change, ≥|1|; FDR, <0.05) from *A. macleodii* ATCC 27126 grown under carbon or iron limitation with corresponding DESeq2 results. Download Data Set S1, XLSX file, 0.3 MB. Copyright © 2020 Manck et al. 2020 Manck et al. This content is distributed under the terms of the Creative Commons Attribution 4.0 International license.

Transcripts enriched under iron limitation include homologs to known iron acquisition pathways. This includes a putative Fe(III) ABCT system and a predicted siderophore biosynthetic gene cluster that exhibited a marked 97-fold increase in transcript abundance under iron limitation. Siderophore biosynthesis by ATCC 27126 was confirmed with a chrome azurol S (CAS) assay (see Text S1 and Fig. S3), the first experimental evidence for the presence and expression of a siderophore production pathway in ATCC 27126 or, indeed, *Alteromonas* spp. Additionally, the transcript encoding a cytoplasmic siderophore-interacting protein (MASE_18235) was enriched 18-fold under iron limitation. A sulfur mobilization (SUF) Fe-S cluster assembly system (MASE_12855 to MASE_12895), shown to be critical for the utilization of certain siderophores by Escherichia coli (30–33), was also significantly enriched. Transcripts depleted under iron limitation include the ferric uptake repressor protein (Fur) (MASE_08050) and transcripts encoding two subunits of a bacterioferritin homolog (MASE_09805 and MASE_09810), an iron storage protein.

10.1128/mSystems.00070-20.3 FIG S3 CAS assay results. (A) No siderophore production was detected for *Alteromonas* sp. strain ALT199, as expected based on a search for known biosynthetic pathways within the genome (Fig. 5). (B) Siderophore production by *Alteromonas macleodii* ATCC 27126 was detected, as indicated by the orange halo extending beyond cells outlined in black. (C) *Ruegeria* sp. strain TM1040 was used as a negative control, and no siderophore production was detected as expected. Download FIG S3, PDF file, 2.7 MB. Copyright © 2020 Manck et al. 2020 Manck et al. This content is distributed under the terms of the Creative Commons Attribution 4.0 International license.

10.1128/mSystems.00070-20.7 TEXT S1 Materials and methods for the preparation of a CAS assay for the detection of siderophore production by ATCC 27126. Download Text S1, PDF file, 0.1 MB. Copyright © 2020 Manck et al. 2020 Manck et al. This content is distributed under the terms of the Creative Commons Attribution 4.0 International license.

**FIG 2:**
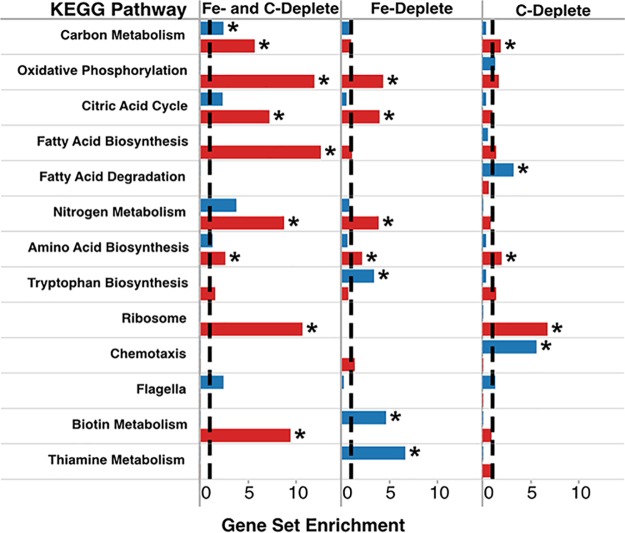
Gene set enrichment analysis of differentially expressed genes assigned to selected KEGG pathways. The results are separated into gene sets based on differential expression under either exclusively carbon-depleted conditions (right column), exclusively iron-depleted conditions (middle column), or under both nutrient-depleted conditions (left column) and whether genes were enriched (fold change, ≥2; FDR, <0.05; blue bars) or depleted (fold change, ≤−2; FDR, <0.05; red bars) under the given limitation. The dashed line in each column indicates the expected 1:1 ratio of differentially expressed genes in a given set compared to the total number of genes in the set across the entire genome. Bars extending beyond the dashed line indicate an overrepresentation of differentially expressed genes in the given gene set, and statistically significant results (hypergeometric test, *P* < 0.05) are marked with an asterisk. See Materials and Methods for details.

In order to determine the effects of nutrient limitation on central metabolic processes in ATCC 27126, a gene set enrichment analysis was performed. Where possible, ORFs were assigned to KEGG pathways and grouped into gene sets according to whether they were differentially expressed under both carbon and iron limitation, exclusively iron limitation, or exclusively carbon limitation (Fig. 2). A given pathway was determined to be enriched (and therefore significantly affected by the given nutrient limitation) if it was overrepresented in a given set of differentially expressed genes compared to the entire genome (see Materials and Methods for details). Iron limitation induced changes in central metabolic processes in ATCC 27126, some of which were unique to iron limitation compared with carbon limitation (Fig. 2). Carbon metabolism, oxidative phosphorylation, and the citric acid cycle were significantly affected by iron limitation (Fig. 2). Specifically, a distinct shift toward the glyoxylate cycle as an alternative pathway to the citric acid cycle (CAC) was observed as evidenced by an enrichment in transcripts for isocitrate lyase (MASE_13040) and malate synthase (MASE_13050). A majority of the enzymes that make up the CAC were depleted under iron limitation; however, there was a marked 20-fold increase in the expression of fumarase C (MASE_14985), the only non-iron containing version of this enzyme. Additionally, the major iron-containing subunits of the electron transport chain (for NADH-ubiquinone reductase, MASE_16205 to MASE_16230; and for ubiquinol-cytochrome *c* reductase, MASE_03300 to MASE_03310; and multiple cytochrome oxidase complexes) were significantly depleted under iron limitation between 2- and 6-fold. A decrease in the transcript abundance of the Entner-Doudoroff (ED) glycolysis pathway (MASE_11170 to MASE_11190) was also observed. Notably, 6-phosphogluconate dehydratase of the ED pathway is an iron-containing metalloprotein. Metabolic pathways that were uniquely affected by iron limitation included enrichment of the tryptophan, biotin, and thiamine biosynthetic pathways (Fig. 2). Notably, a nickel-containing superoxide dismutase (MASE_02360) was significantly enriched under iron limitation, possibly reducing the iron requirements associated with oxidative stress (34, 35).

The global transcriptomic response of ATCC 27126 to carbon limitation was very distinct compared to that of iron limitation (Fig. 1). Components of central carbon metabolism, such as the CAC and ED pathway, were similarly depleted (Fig. 2), but upregulation of the glyoxylate cycle was not observed. Fatty acid degradation was uniquely affected by carbon limitation (Fig. 2). Transcripts required for fatty acid oxidation (MASE_02310, MASE_02315, MASE_03100, and MASE_10260) were upregulated under carbon limitation up to 3-fold, perhaps indicating a shift to alternative carbon sources in the absence of glucose. A particularly striking feature of the carbon-limited transcriptome that was absent from the iron limited transcriptome was the significant enrichment of chemotactic response systems, flagellar biosynthesis, and agglutinin production (Fig. 2). In addition to components of central carbon metabolism, transcripts involved in fatty acid biosynthesis, nitrogen metabolism, and ribosome production were all significantly depleted under both iron and carbon limitation (Fig. 2).

### Differential expression of TBDTs under nutrient limitation.

**FIG 3:**
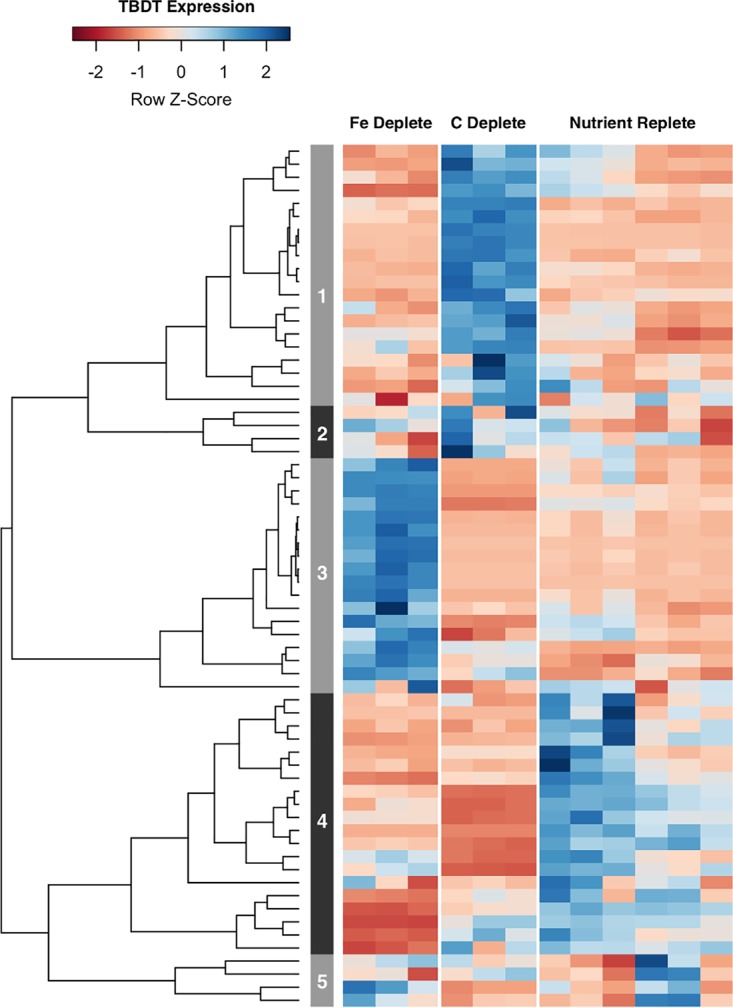
Heat map displaying normalized transcript counts of identified TBDTs in the Alteromonas macleodii ATCC 27126 genome under iron-depleted (*n *= 3), carbon-depleted (*n *= 3), and nutrient-replete (*n *= 6) conditions. Individual columns within each nutrient category display results for a single replicate. Each row displays results for a single TBDT, with coloring depicting expression levels under each nutrient condition. Transcripts with higher-than-average expression levels are displayed in blue, while those with lower-than-average expression are displayed in red. The dendrogram displays hierarchical clustering results based on expression patterns of TBDT transcripts across all nutrient conditions. The numbered sidebar depicts clusters grouped together at two-thirds of the maximum dendrogram height. See Data Set S2 for a complete summary of statistically significant TBDT differential expression.

Sixty-six putative TBDTs are transcribed by the genome of *A. macleodii* ATCC 27126. Using the same significance threshold of a ≥2-fold change and FDR of <0.05, transcripts for 11 TBDTs were found to be significantly enriched under iron limitation, while 15 TBDTs were enriched under carbon limitation without overlap. Expression patterns for iron-responsive and carbon-responsive TBDTs are clearly distinct from each other, as well as the remaining TBDTs (Fig. 3). Overall, transcripts for TBDTs were some of the most highly enriched transcripts in the genome under either nutrient limitation. See Data Set S2 for a complete summary of TBDT differential expression.

10.1128/mSystems.00070-20.9 DATA SET S2 Gene loci of the 66 putative TBDTs transcribed by *A. macleodii* ATCC 27126 and the statistically significant differential expression of each transcript under iron or carbon limitation (log_2_ fold change, ≥|1|; FDR, <0.05). n.s., conditions where no significant differential expression of the transcript was detected under a given limitation. Download Data Set S2, XLSX file, 0.1 MB. Copyright © 2020 Manck et al. 2020 Manck et al. This content is distributed under the terms of the Creative Commons Attribution 4.0 International license.

### Gene neighborhood analysis of Fe-responsive and C-responsive TBDTs.

**FIG 4:**
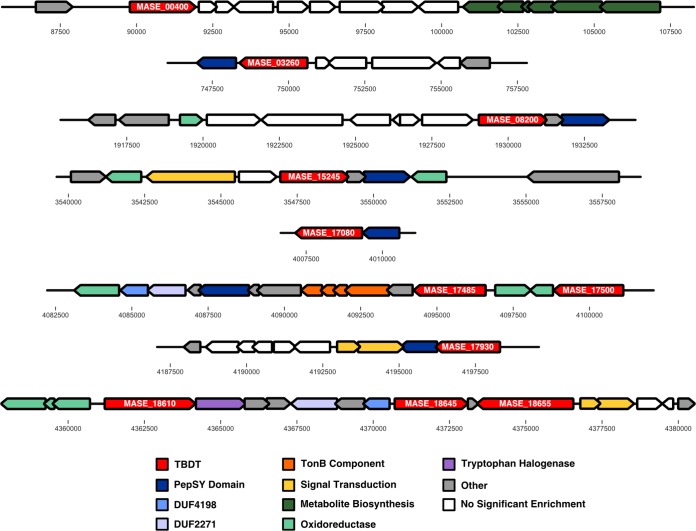
Gene neighborhoods of TBDTs enriched under iron limitation. Arrows represent the gene length and arrangement on the chromosome. Colored genes were found to be enriched in transcript abundance under iron limitation (fold change, ≥2; FDR, <0.05), and colors represent putative function as assigned in the legend based on detected conserved domains. White genes showed no enrichment under iron limitation. Genes encoding TBDTs are labeled with gene loci referenced in the text.

We investigated the gene neighborhoods of the 26 carbon- or iron-regulated TBDTs in an effort to further elucidate the corresponding substrates for these transporters. (Fig. 4 and S4). The gene neighborhoods of six of the Fe-responsive TBDTs include genes containing a PepSY-associated transmembrane domain (pfam03929), each also significantly enriched under iron limitation (Fig. 4). The proteins encoded by these genes are predicted to contain between 4 and 8 transmembrane helices. An additional gene with this conserved domain and predicted topology (MASE_09745) is found within the siderophore biosynthetic gene cluster of ATCC 27126, further linking this domain to a role in iron transport. Proteins with similar topology and the PepSY transmembrane domain have been characterized as the inner membrane permeases for the uptake of the siderophores pyochelin and ferrichrome in Pseudomonas aeruginosa and rhizobactin 1021 in Sinorhizobium meliloti in place of a classical ABCT (36–38). Thus far, there is limited sequence identity between characterized permeases of this type, making homologous genes difficult to detect. However, the protein topology, the presence of a PepSY transmembrane helix, and the colocation with an Fe-regulated TBDT allow us to strongly predict that these genes encode inner membrane permeases for the transport of at least six distinct siderophores by ATCC 27126. The presence of these PepSY inner membrane permeases also helps explain the mismatch between the number of TBDTs in ATCC 27126 and the number of ABCTs for transport across the inner membrane. With far fewer ABCTs than TBDTs (none of which are associated with an Fe-responsive TBDT), ATCC 27126 must either utilize ABCTs for the nonspecific transport of multiple substrates, or there must be alternative methods of transport across the inner membrane that are not well described for marine bacteria. The identification of these PepSY permeases in ATCC 27126 lends support to the latter mechanism.

10.1128/mSystems.00070-20.4 FIG S4 Gene neighborhoods of TBDTs enriched under carbon limitation in ATCC 27126. Arrows represent gene length and arrangement on chromosome. Filled, colored genes were found to be enriched in transcript abundance under carbon limitation (fold change, ≥2; FDR, <0.05), and colors represent putative function as assigned in legend based on detected conserved domains. White genes showed no enrichment under carbon limitation, but colored outlines depict putative function as assigned in the legend. Genes encoding TBDTs are labeled with gene loci as referenced in text. AE, aldose 1-epimerase; GALT, galactose-1-phosphate uridylyltransferase; GALK, galactokinase; GK, glycerol kinase; GPDH, glycerol-3-phosphate dehydrogenase; PHB, polyhydroxybutyrate; GPDA, N-acetylglucosamine-6-phosphate deacetylase; GFPT, glucosamine-fructose-6-phosphate aminotransferase; GLUK, glucosamine kinase; XR, d-xylose reductase; GH, glycosyl hydrolase; ACE, acetylesterase; XI, xylose isomerase; XK, xylulokinase; SBP, solute binding protein. Download FIG S4, PDF file, 0.6 MB. Copyright © 2020 Manck et al. 2020 Manck et al. This content is distributed under the terms of the Creative Commons Attribution 4.0 International license.

**FIG 5:**
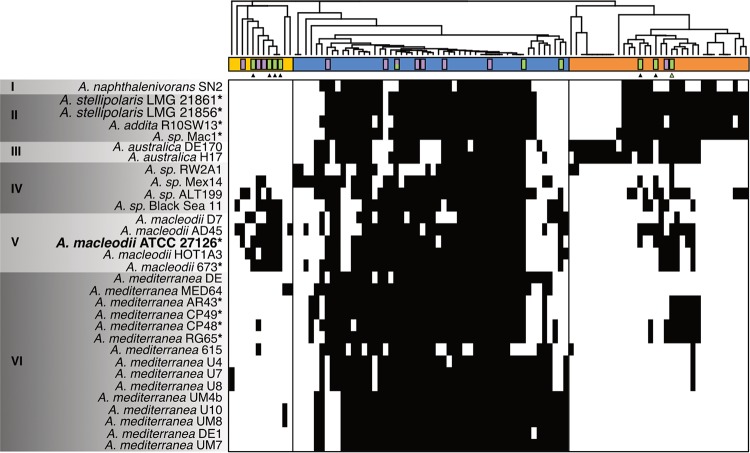
Distribution of TBDT clusters across the *Alteromonas* genus. Strains are ordered according to the phylogeny of López-Pérez et al. (85). Roman numerals indicate strains belonging to the same species, with the exception of group IV, which groups strains unidentified at the species level and which share less than 95% average nucleotide identity with any other strain. Alteromonas macleodii ATCC 27126, used in this study, is in bold. All strains with predicted siderophore biosynthetic gene clusters are noted with an asterisk. Columns represent distinct TBDT clusters with two or more nodes determined from Fig. S5 and Data Set S3. The presence of one or more TBDTs from a given strain in each cluster is denoted by a filled rectangle in the heat map. The dendrogram displays the hierarchical clustering of this distribution across the genus, where the number of TBDTs from a given strain in each cluster was normalized to the total number of TBDTs in the cluster. The yellow, blue, and orange sidebars distinguish the three major divisions in the dendrogram. Purple bars denote clusters with C-responsive TBDTs in Alteromonas macleodii ATCC 27126 (fold change, ≥2; FDR, <0.05), and green bars denote clusters with Fe-responsive TBDTs (fold change, ≥2; FDR, <0.05). The split bar denotes the largest cluster from Fig. S5, which contains both Fe- and C-responsive TBDTs. Triangles indicate predicted siderophore TBDT clusters, and the green triangle specifically indicates the predicted cluster for the cognate TBDT of the endogenous siderophore.

Gene neighborhood analysis has also revealed putative regulatory mechanisms associated with two of the Fe-responsive TBDTs (Fig. 4), providing preliminary evidence of a more nuanced regulatory system beyond Fur regulation of iron transport. MASE_18660 and MASE_18665 show homology to the FecIR regulatory system for the transport of ferric citrate, as characterized in Escherichia coli (39–41), indicating that the associated TBDT (MASE_18655) is responsible for the transport of ferric citrate. MASE_17915 and MASE_17920 show homology to the pfeRS regulatory system identified in P. aeruginosa for the transport of the siderophore enterobactin (42, 43). The TBDT associated with this regulatory system in ATCC 27126 (MASE_17930) is present in all strains across the *Alteromonas* genus that also possess the same siderophore biosynthetic gene cluster as ATCC 27126 (Fig. 5). MASE_17930 is also colocated with a PepSY permease, as described above. This indicates that MASE_17930 may be responsible for the transport of the endogenous siderophore produced by ATCC 27126.

10.1128/mSystems.00070-20.5 FIG S5 Sequence similarity network of TBDT peptide sequences from 31 *Alteromonas* strains. Each node represents a single sequence, and clusters are defined with an alignment score threshold of 110. Enlarged nodes colored purple were enriched in Alteromonas macleodii ATCC 27126 under carbon limitation (fold change, ≥2; FDR, <0.05), and likewise, those colored green were enriched under iron limitation (fold change, ≥2; FDR, <0.05). See Data Set S3 for corresponding gene loci within each cluster. Download FIG S5, PDF file, 1.0 MB. Copyright © 2020 Manck et al. 2020 Manck et al. This content is distributed under the terms of the Creative Commons Attribution 4.0 International license.

10.1128/mSystems.00070-20.10 DATA SET S3 Gene loci of the 1,804 putative TBDTs from 31 *Alteromonas* strains and corresponding cluster location in the sequence similarity network (Fig. S5). Clusters are numbered from left to right and top to bottom in Fig. S5. Singlet clusters labeled together as cluster 98. Download Data Set S3, XLSX file, 0.1 MB. Copyright © 2020 Manck et al. 2020 Manck et al. This content is distributed under the terms of the Creative Commons Attribution 4.0 International license.

A final notable feature of the gene neighborhoods of the Fe-responsive TBDTs in ATCC 27126 is the presence of gene pairs containing the conserved domains of unknown function DUF4198 (pfam10670) and DUF2271 (pfam10029). These domains were identified exclusively in the neighborhoods of the Fe-responsive TBDTs and are themselves also enriched under iron limitation (MASE_17430, MASE_17435, MASE_18630, and MASE_18640) (Fig. 4). Three of these four genes have a high likelihood (>0.70) of a leading signal peptide, implying that they are likely functioning as either periplasmic or outer membrane-associated proteins. The DUF4198 domain has been identified in components of the energy-coupling factor-type ABCTs for nickel and cobalt, where they are thought to couple the transmembrane movements of additional components in these systems (44, 45). The DUF2271 domain falls within the cl21544 superfamily, characterized by an immunoglobulin-like β-sandwich fold indicating a possible ligand or protein interaction site. DUF2271 is found at the C-terminal ends of MASE_17435 and MASE_18630, each of which also contains an N-terminal domain that associate them with membrane-anchored proteins (pfam16357 and COG1477, respectively). Given the novelty of these domains and their specific association with Fe-responsive TBDTs, further characterization of these proteins is warranted.

The 15 TBDTs enriched under carbon limitation also have unique gene neighborhoods which are often composed of ancillary genes for the catabolism of complex polymers (Fig. S4). Adjacent catabolic genes include predicted β-glucosidase (MASE_19120 and MASE_19670), α-glucosidase (MASE_13325), α-galactosidase (MASE_00935), α-xylosidase (MASE_18035), α-*N*-arabinofuranosidase (MASE_19120), β-*N*-acetylhexosaminidase (MASE_13870), polyhydroxybutyrate (PHB) depolymerase (MASE_06600), and peptidases (MASE_03530, MASE_10030, and MASE_12160). This would suggest the ability of ATCC 27126 to acquire and grow on α- and β-glucans, xyloglucans, α-galactosides, α-arabinosides, *N*-acetyl-β-d-hexosaminides, PHB, and proteins. In contrast to the neighborhoods of the Fe-responsive TBDTs, a majority of the neighboring genes of the C-responsive TBDTs were not also enriched under carbon limitation. This suggests that additional regulatory mechanisms may be at play for the expression of the corresponding hydrolytic enzymes. Indeed, a majority of the gene neighborhoods of the C-responsive TBDTs contain a transcriptional regulator, which may act as a positive transcriptional regulator of catabolism-related genes should the target compound be transported (Fig. S4).

Table 1 summarizes the transcriptomic response and predicted substrate for each of the TBDTs enriched under either carbon or iron limitation in this data set. It is worth noting that heme and hemoproteins are an additional class of Fe-containing compounds that serve as sources of iron in the marine environment (46, 47) and are acquired via TBDTs in Gram-negative bacteria. They are therefore possible candidates for the substrates of the remaining Fe-responsive TBDTs. However, we did not find evidence of a complete heme transport system in ATCC 27126 as characterized in other marine bacteria (48, 49). Notably, ATCC 27126 lacks a heme-specific ABCT. Nevertheless, given the evidence for alternative mechanisms of inner membrane transport for siderophores in ATCC 27126, we cannot rule out the possibility that heme is likewise transported across the inner membrane via a nontraditional mechanism and that one or more of the remaining Fe-responsive TBDTs are responsible for heme acquisition. While experimental validation using gene disruption and phenotyping is necessary to confirm all of these putative annotations, we have effectively generated a set of target transport systems to be further tested in this genetically tractable organism (50, 51).

**TABLE 1:** Gene loci of Fe- and C-responsive TBDTs in *A. macleodii* ATCC 27126

TBDT gene locus	Limitation	Log_2_ fold change	Predicted substrate^*a*^
MASE_00400	Fe	2.78	—
MASE_03260	Fe	1.19	Exogenous siderophore
MASE_08200	Fe	2.54	Exogenous siderophore
MASE_15245	Fe	2.24	Exogenous siderophore
MASE_17080	Fe	1.55	Exogenous siderophore
MASE_17485	Fe	5.78	Exogenous siderophore^*b*^
MASE_17500	Fe	3.88	Exogenous siderophore^*b*^
MASE_17930	Fe	4.51	Endogenous siderophore
MASE_18610	Fe	1.99	—
MASE_18645	Fe	6.95	—
MASE_18655	Fe	1.12	Ferric citrate
MASE_00935	C	1.35	Galactolipids
MASE_03530	C	3.28	Peptide
MASE_04115	C	1.28	—
MASE_05185	C	1.00	—
MASE_05565	C	1.50	—
MASE_06600	C	1.59	Polyhydroxybutyrate
MASE_08095	C	4.96	—
MASE_10030	C	1.52	Peptide
MASE_12160	C	2.20	Peptide
MASE_13325	C	5.14	α-Glucans
MASE_13870	C	2.82	*N*-Acetyl-β-d-glucosaminides
MASE_15330	C	1.06	—
MASE_18035	C	1.44	Xyloglucans
MASE_19120	C	1.11	α-Arabinosides
MASE_19670	C	1.66	β-Glucans

a The log_2_ fold changes of TBDT transcripts under nutrient-depleted (*n *= 3) compared to nutrient-replete (*n *= 3) conditions are given along with the predicted substrate, where applicable. A dash (—) indicates that a substrate prediction for a given TBDT was not possible, based on gene neighborhood analysis.

b MASE_17485 and MASE_17500 are located with each other as well as with a single gene containing a PepSY transmembrane domain (Fig. 4). Therefore, the association of the PepSY domain with either of those TBDTs (and therefore the putative annotation as a siderophore transporter) is ambiguous.

### Sequence similarity network of TBDTs across the genus *Alteromonas*.

In order to evaluate the distribution of these TBDTs across the genus *Alteromonas*, we performed a clustering analysis based on the sequence alignment of all TBDTs detected in the genomes of 31 strains of *Alteromonas*. This grouped 1,804 peptide sequences into 97 distinct clusters containing two or more sequences (Fig. S5 and Data Set S3). While some clusters within the network contain multiple TBDTs from a single strain that are challenging to resolve, a majority of the clusters separate into distinct groups, where each cluster presumably resolves a coherent group of transporters. Further hierarchical clustering of these distinct groups of TBDTs shows that a core group of TBDTs are present across most *Alteromonas* strains (Fig. 5, blue bar), while the remaining two groups have a patchy distribution (Fig. 5, yellow and orange bars). Notably, Alteromonas mediterranea lacks many TBDTs beyond the core group, and this is not due to a reduced genome size in this species (Fig. S6).

10.1128/mSystems.00070-20.6 FIG S6 Linear regression between the total number of TBDTs per genome and the total protein-coding genes per genome for 31 strains in the *Alteromonas* genus. Data points for each strain are colored according to the species groups defined in Fig. 5. There is no significant trend between the total number of TBDTs per *Alteromonas* genome and the total number of protein-coding genes per genome. Download FIG S6, PDF file, 0.2 MB. Copyright © 2020 Manck et al. 2020 Manck et al. This content is distributed under the terms of the Creative Commons Attribution 4.0 International license.

When the Fe-responsive TBDTs from ATCC 27126 are mapped onto this distribution, an interesting pattern emerges. MASE_18610, MASE_18645, MASE_18655, and MASE_00400 fall within the core group of TBDTs that are widely distributed across the *Alteromonas* genus. Based on our functional analysis, none of these TBDTs are predicted to transport iron-siderophore complexes (Table 1). Rather, the distribution of the predicted iron-siderophore transporters from ATCC 27126 is constrained to fewer taxa (Fig. 5). In particular, all of the predicted receptors for exogenous siderophore complexes in ATCC 27126 are absent from *A. mediterranea*. However, several strains of *A. mediterranea* do have a predicted siderophore biosynthesis pathway and the predicted cognate receptor. In contrast, a majority of the C-responsive TBDTs from ATCC 27126 fall within the group most widely distributed across *Alteromonas* spp. However, four C-responsive TBCTs (MASE_10030, MASE_13870, MASE_15330, and MASE_18035) are found outside this group and are therefore largely absent from *A. mediterranea*.

## DISCUSSION

### Metabolic responses to iron and carbon limitation are distinct.

Limitations of iron and carbon in heterotrophic bacteria are typically studied separately. Remarkably, despite the shared aspect of growth limitation, iron and carbon limitation resulted in distinct reorganizations of cellular metabolism and nutrient acquisition in *A. macleodii* ATCC 27126. Iron limitation was characterized by a decreased dependence on iron-containing proteins, while cellular resources were directed toward iron acquisition. Fe-sparing mechanisms included a downregulation of the major Fe-containing enzymes of the CAC, ED glycolysis, and electron transport chain with a corresponding upregulation of Fe-lacking metabolic replacements (nickel superoxide dismutase [Ni-SOD] and fumarase C). A shift to the glyoxylate cycle as an alternative to the CAC was also observed in this work and has been observed under iron limitation for two additional strains of *Alteromonas* (52). An increase in the expression of isocitrate lyase has also been observed in the gammaproteobacterium Photobacterium angustum under iron limitation (53). Here, the authors were able to demonstrate decreased growth rates and respiration under iron limitation in a knockout mutant lacking isocitrate lyase compared to the wild type, suggesting that this pathway is an effective mechanism for coping with iron limitation (53). However, this transition to the glyoxylate cycle has also been observed in a range of taxa as a response to multiple different nutrient limitations as well as oxidative stress and is unlikely to be an iron-specific response (54–56). Similar cell-wide responses to iron limitation have been observed for additional *Proteobacteria* strains (57, 58). This work demonstrates that these responses extend to the marine environment and reinforces the control that iron availability may have on carbon metabolism in heterotrophic bacteria.

Under carbon limitation, there was no observed switch to the glyoxylate pathway. However, components of the CAC along with upstream pathways for glucose processing were similarly depleted, likely driven by the lack of glucose as a starting substrate. In striking contrast to iron limitation, carbon scavenging was seemingly enhanced by an increase in motility and particle formation as a result of a dramatic upregulation of chemotactic response systems as well as flagellar biosynthesis and agglutinin production. An increase in motility has been observed under changing environmental conditions in additional strains of *Alteromonas* (59, 60). This has been observed under growth in minimal medium (59) as well as in transition to copiotrophic growth upon phytoplankton decay and the input of organic matter (60). An increase in motility under both copiotrophic and carbon-limited growth may suggest differing regulatory pathways governing these responses. Under copiotrophic growth, this was attributed to a multifaceted response involving the small RNA-binding protein CsrA and its activation of the FliA sigma factor (60). In the current study, we did not observe differential expression of either *csrA* or *fliA*, highlighting the complexity of motility regulation in *Alteromonas* spp. Furthermore, the lack of a motility response under iron limitation is intriguing and may suggest that this strategy quickly became too energetically expensive under these conditions.

### Transporters inform our view of the chemical matrix of the marine environment.

When considering the specific response to iron and carbon limitation of the 66 putative TBDTs found in ATCC 27126, we find that ATCC 27126 uses a distinct set of TBDTs to acquire a wide range of iron and carbon complexes (Table 1). These results emphasize the importance of experimental evidence for validating TBDT annotations. Each of these sets responds strongly and exclusively to the corresponding nutrient limitation. This indicates that there is a high degree of regulation tuning the expression of these transporters to specific environmental conditions. For the Fe-responsive TBDTs, this is likely primarily controlled by Fur regulation; however, we have also found preliminary evidence of regulatory systems that may respond to the presence of specific iron-ligand complexes. In contrast, carbon acquisition appears to be primarily controlled by a substrate-specific response. While the TBDT components of these systems were broadly sensitive to carbon limitation, the expression of hydrolytic enzymes and supporting proteins may depend on the presence of a given substrate, much like the canonical *lac* operon. This is supported by the identification of transcriptional regulatory systems in a majority of these gene neighborhoods. These results also highlight that the transport of substrates across the inner membrane of ATCC 27126 is likely accomplished by a diverse set of mechanisms. We have detected 6 putative inner membrane permeases with a PepSY domain that show significant structural similarity to characterized permeases for the transport of siderophores in well-studied pathogenic bacteria (36–38). To our knowledge, this is the first description of such permeases in a marine bacterium and provides a new, highly specific target for understanding the role of siderophore utilization in the marine environment.

One of the most notable aspects of this data set is not only the total number of putative TBDTs found in ATCC 27126, but also the similarity in magnitude between the number of iron-responsive and carbon-responsive TBDTs. This implies that the diversity of organic carbon and iron complexes encountered and utilized by ATCC 27126 in its environment must be of a similar scale. Particularly regarding iron-ligand complexes, relatively few compounds have been isolated and structurally characterized directly from seawater. Even for the case of siderophores, where there has been a diverse range of structures isolated from cultured marine strains (61), only a small subset have been detected in natural seawater (8, 62, 63). Given these analytical challenges, the ability to test the bioavailability of relevant complexes in the marine environment has been limited. By focusing on cellular transport systems, this work has suggested an unexpected diversity of complex iron and carbon substrates that are bioavailable in the marine environment. Further characterization of these transport systems, both within *Alteromonas* spp. and additional taxa, will continue to inform our understanding of the bioavailable iron and carbon pools in the marine environment.

### Niche specialization drives differentiation in the distribution of TBDTs.

Across sequenced representatives of the *Alteromonas* genus, there is a high capacity for transport via TBDTs, with strains possessing between 38 and 76 putative TBDTs (Fig. S6). However, across the genus, the distribution of specific TBDTs is not uniform, particularly with regard to predicted iron transporters (Fig. 5). While a majority of the C-responsive TBDTs in ATCC 27126 are widely distributed across *Alteromonas* spp., many of the Fe-responsive TBDTs, particularly those for the predicted transport of exogenous siderophores, were constrained to fewer taxa and notably absent from *A. mediterranea*. This may indicate that exogenous siderophore utilization (i.e., a “cheater” strategy [64]) is niche specific, or perhaps that distinct sets of siderophores prevail in different niches.

Phylogenetic randomness in the distribution of trace metal transporters within two lineages of marine *Alphaproteobacteria* has been described, which the authors interpret as trace metal niche differentiation (65). Additionally, in an evaluation of the pangenome of *Alteromonas* spp., López-Pérez and Rodriguez-Valera (66) find that even between strains of a single *Alteromonas* species, genes encoding transporters, including TBDTs, exhibited some of the highest *dN*/*dS* ratios. High *dN*/*dS* ratios indicate positive selective pressure on these genes and support the idea of niche specialization in substrate utilization. Specifically, trace metal availability in a given environment may impart a significant control on genomic content, differentiating phylogenetically similar strains.

Compared to other well-studied marine copiotrophic bacteria, the results presented here fit along an emerging spectrum of strategies for substrate utilization that ranges from the dedicated transport of monomeric substrates through ABCTs to the use of TBDTs for the acquisition of large, complex compounds. It has been suggested that substrate availability, as dictated by the use of either TBDTs or ABCTs by a given taxonomic group, creates distinct ecological niches and can be a primary control on the succession of heterotrophs during a phytoplankton bloom (67–69). For example, flavobacteria, whose genomes are enriched in TBDTs and hydrolytic enzymes, are known for their ability to break down and acquire high-molecular-weight DOM (70, 71) and therefore dominate during the peak and decay stages of a bloom (72, 73). In contrast, well-studied roseobacters, with large numbers of ABCTs, specialize in the transport of low-molecular-weight DOM (65, 74). This can be released following the breakdown of high-molecular-weight DOM or directly from phytoplankton during early bloom stages. Previous studies have highlighted the potential utilization of complex substrates by *Alteromonas* spp., particularly with regard to carbon acquisition (23, 26, 28, 75). With the identification of hydrolytic enzymes and associated C-responsive TBDTs in ATCC 27126, this work further supports the conclusions that members of the *Alteromonas* genus contribute significantly to the degradation of complex marine DOM. Furthermore, we provide the first evidence suggesting that *Alteromonas* spp. are likewise enriched in cellular machinery for the transport of larger, organically complexed iron substrates.

Given the significant role that iron plays in carbon metabolism in heterotrophic bacteria, understanding the molecular transport mechanisms and resulting bioavailability of both of these nutrients will important steps in understanding the turnover of organic matter in the marine environment. While a majority of previous work has focused on the acquisition of carbon by heterotrophic bacteria, the results presented here show that for *Alteromonas* spp., the transport of large, complex substrates holds true for both carbon and iron acquisition. However, the relationship between the substrate-specific bioavailability of iron and carbon remains to be explicitly tested for many copiotrophic taxa. It is likely that the interplay of the acquisition of these two nutrients by different taxa of heterotrophic bacteria will underlie dynamics such as ecological succession and ultimately affect the balance between export and recycling in a given marine environment.

## MATERIALS AND METHODS

### Bacterial strains and growth conditions.

The *A. macleodii* type strain ATCC 27126 was used for all growth experiments. Medium was prepared and cultures grown using appropriate aseptic techniques. Cultures were maintained on Marine broth (MB) 2216 (BD Difco, Sparks, MD, USA) agar plates and grown in liquid MB 2216 to initiate experiments. Limitation experiments as described below were conducted in peptone-casein medium with glucose as a carbon source (PC+) and peptone-casein medium with no added carbon source (PC). The medium was prepared by mixing 500 ml of 0.2-μm-filtered seawater collected from the Scripps Institution of Oceanography Pier with 0.5 g casein, 0.5 g bacteriological peptone, and 0.126 g NH_4_Cl. For the PC^+^ medium, 1.8 g of glucose was also supplied. The resulting solution was adjusted to pH 7.6, microwave sterilized, and stirred overnight with 7% (wt/vol) Chelex 100 resin (Bio-Rad, Hercules, CA, USA) to remove contaminating trace metals. These base medium solutions were again filtered through a 0.2-μm filter to remove the Chelex resin and microwave sterilized an additional time. A trace-metal master mix solution (4.0 × 10^−5^ M ZnSO_4_, 2.3 × 10^−4^ M MnCl_2_, 2.5 × 10^−5^ M CoCl_2_, 1.0 × 10^−5^ M CuSO_4_, 1.0 × 10^−4^ M Na_2_MoO_4_, 1.0 × 10^−5^ M Na_2_SeO_3_) was prepared in 0.1 M HCl. A 0.05 M Na_2_EDTA·2H_2_O solution was prepared in Milli-Q·H_2_O and adjusted to pH 8. Finally, a 0.735 M KH_2_PO_4_ solution in Milli-Q·H_2_O was also prepared. The trace-metal mix, EDTA, and KH_2_PO_4_ solutions were 0.2-μm-filter sterilized and each diluted 1,000-fold into aliquots of the appropriate base medium immediately before inoculation with *A. macleodii* ATCC 27126. A 5 × 10^−3^ M FeCl_3_·6H_2_O solution was also prepared in 0.1 M HCl, 0.2-μm-filter sterilized, and diluted 1,000-fold into aliquots of the base media for iron-replete cultures.

### Iron- and carbon-limited growth experiments.

*A. macleodii* ATCC 27126 was maintained on MB 2216 agar, and triplicate colonies were inoculated in 5 ml of liquid MB 2216 to initiate growth experiments. All liquid cultures were maintained in the dark at room temperature with shaking at 190 rpm. After 8 h of growth, 100 μl of each liquid MB 2216 culture was then transferred to 5 ml of PC^+^ medium without any added iron source. The triplicate cultures were allowed to grow for 12 h in order to reach an iron-limited state, and 100 μl of each culture was then transferred to two 5-ml aliquots of fresh PC^+^ medium, one with no added iron source (iron depleted) and the other with an added 5 × 10^−6^ M FeCl_3_ as the iron source (iron replete). The synthetic ligand EDTA was added at a final concentration of 5.0 × 10^−5^ M in order to buffer the free iron concentration. This results in a calculated free iron concentration of 6.5 × 10^−9^ M for replete cultures. The cultures were allowed to grow for 6.5 h until mid-exponential phase was reached in the iron-replete cultures (average OD_600_, 0.68; Fig. S1). Cells were then harvested for RNA extraction, as described below. Carbon-limited cultures were grown in a similar manner with the use of PC medium lacking glucose as the primary carbon source to achieve a carbon-limited state. All carbon-limited cultures were maintained under iron-replete conditions (5 × 10^−6^ M FeCl_3_), and the final experimental cultures were grown in contrasting PC (carbon-depleted) and PC^+^ (carbon-replete) media.

### Library preparation and sequencing.

When replete cultures were in mid-exponential phase, cells were pelleted via centrifugation at 6,000 × *g* for 5 min at 4°C. The resulting pellet was resuspended in 200 μl Max bacterial enhancement reagent (Ambion, Carlsbad, CA, USA) preheated to 94°C and incubated for 4 min. One milliliter of TRIzol reagent (Ambion) was added, and total RNA was extracted using the Direct-zol RNA miniprep kit (Zymo Research, Irvine, CA, USA), according to the manufacturer’s protocol. Contaminating DNA was removed with the provided DNase solution. Sequencing libraries were prepared for directional RNA sequencing (RNA-seq) using the WaferGen PrepX kit (TaKaRa Bio, Kusatsu, Japan) and sequenced on the NextSeq 500 platform (2 × 150-bp paired-end reads; Illumina, San Diego, CA, USA). Twenty million to 30 million reads per sample were generated.

### Sequence processing, bioinformatic analysis, and visualization.

Adapters were trimmed from raw sequences using AdapterRemoval (v2.1.7). Quality trimming was performed using Trimmomatic (v0.36). Paired-end reads were mapped with BWA (76) to the reference genome, and read pairs were counted for each gene using featureCounts (v1.6.1). Reads that mapped to protein-coding regions were used for downstream processing. Differential expression of detected open reading frames (ORFs) under various experimental conditions was assessed using DESeq2 (77). Differential expression was considered significant if the fold change between the nutrient-depleted and replete experimental conditions was ≥|2| and the FDR was <0.05 (Benjamin-Hochberg adjusted *P* value).

Gene set enrichment was then tested for ORFs that were differentially expressed under at least one nutrient limitation. Gene set categories were chosen from the Kyoto Encyclopedia of Genes and Genomes (KEGG) pathways. Enrichment was calculated by comparing the number of ORFs assigned to a given KEGG pathway in a group of differentially expressed ORFs compared to the number of ORFs assigned to the same pathway across the entire genome. Enrichment probabilities were then calculated with a hypergeometric test (phyper in R) to determine statistical significance (*P* < 0.05).

Where indicated, functional predictions for ORFs were assigned based on specific hits to conserved domains detected with an RPS-BLAST alignment against the NCBI Conserved Domain Database (78). Protein secondary structure was predicted using the Phyre2 server for protein modeling (79). SignalP (v5.0) was used to predict signal peptides where noted (80).

Genomic neighborhoods were visualized for Alteromonas macleodii accession number CP003841.1. We implemented genomic_neighborhoods.py as a versatile command-line interface built upon DNA Features Viewer (v1.0.0) (81) to seamlessly plot gene neighborhood figures not limited to any particular accession number or species. Our command-line tool is open sourced via GitHub (https://github.com/jolespin/genomic_neighborhood).

### Construction of TBDT sequence similarity network.

The genomes of all available strains within the *Alteromonas* genus were searched for the TBDT conserved barrel domain (pfam00593) using the Joint Genome Institute’s Integrated Microbial Genomes database (82). This resulted in 1,804 peptide sequences obtained from 31 total strains. These sequences were compared in an all-versus-all BLAST analysis using the Enzyme Function Initiative-Enzyme Similarity Tool (83). The resulting sequence similarity output was then organized into clusters with Cytoscape v3.2.1 (84) using an alignment score threshold cutoff of 110.

### Data availability.

All sequence data are openly available under NCBI BioProject number PRJNA591216. Our command-line genomic neighborhood tool is open-sourced via GitHub (https://github.com/jolespin/genomic_neighborhood).
