# Supplementary figures and images for: Transcriptomic Study of Substrate-Specific Transport Mechanisms for Iron and Carbon in the Marine Copiotroph Alteromonas macleodii
Source: mSystems. 2020 Apr 28;5(2):e00070-20. doi: 10.1128/mSystems.00070-20 (PMC7190382; doi:10.1128/mSystems.00070-20)

Figure S1

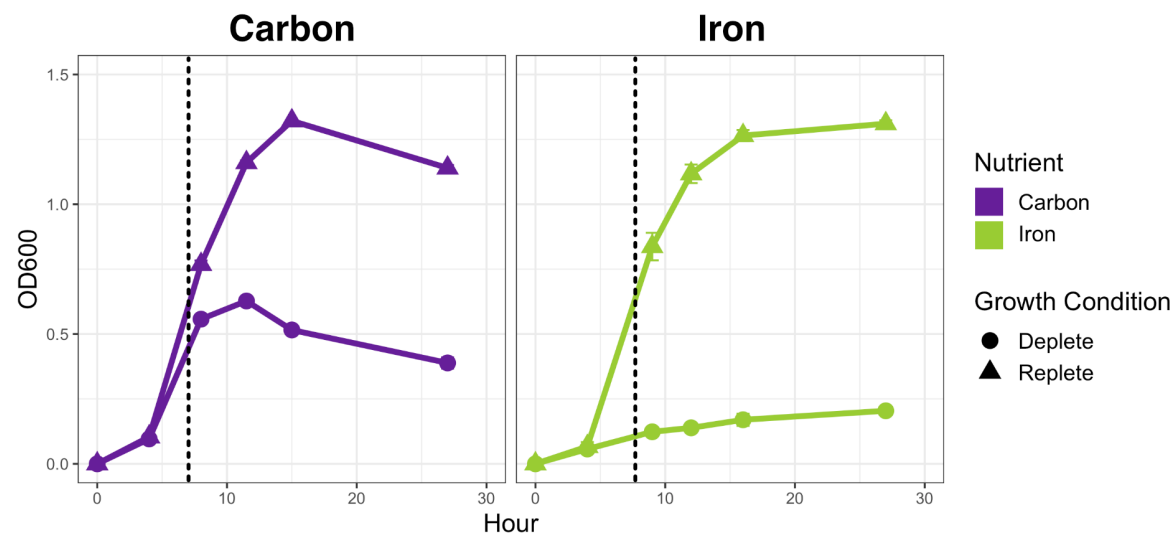

Supplement: FIG S1 [file mSystems.00070-20-sf001.pdf]

**Figure S2**

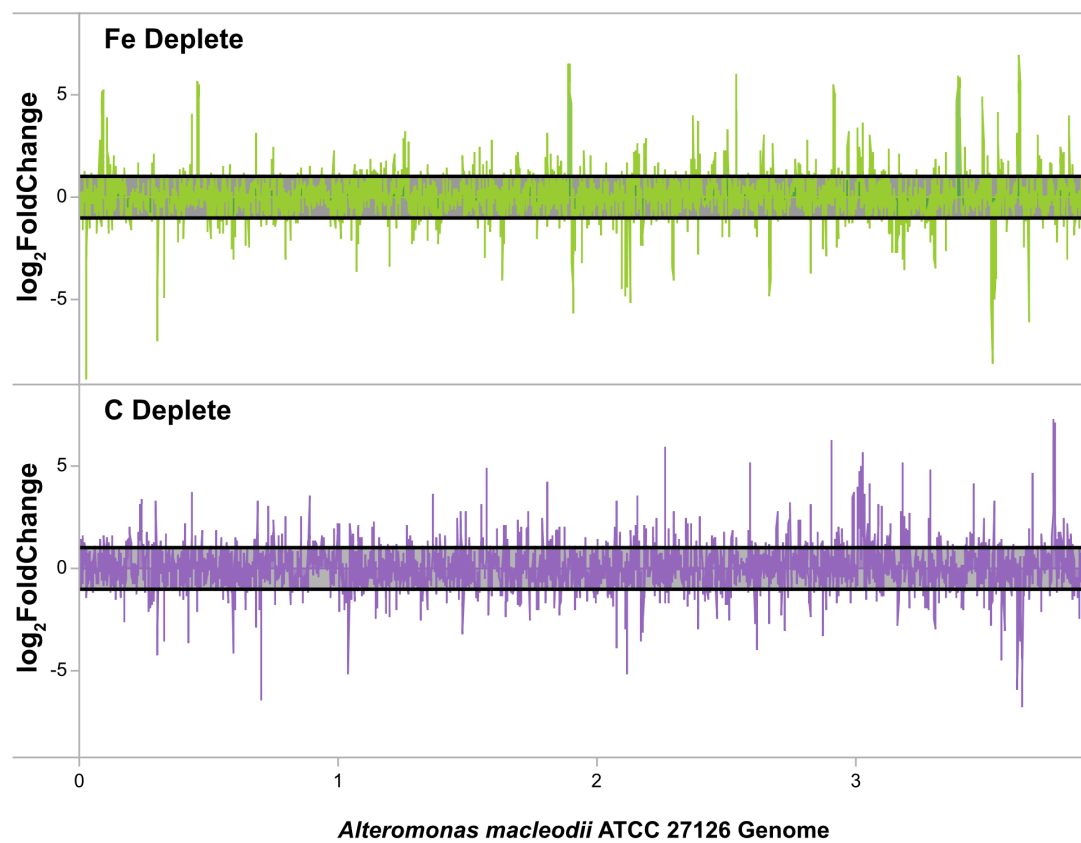

Supplement: FIG S2 [file mSystems.00070-20-sf002.pdf]

**Figure S3**

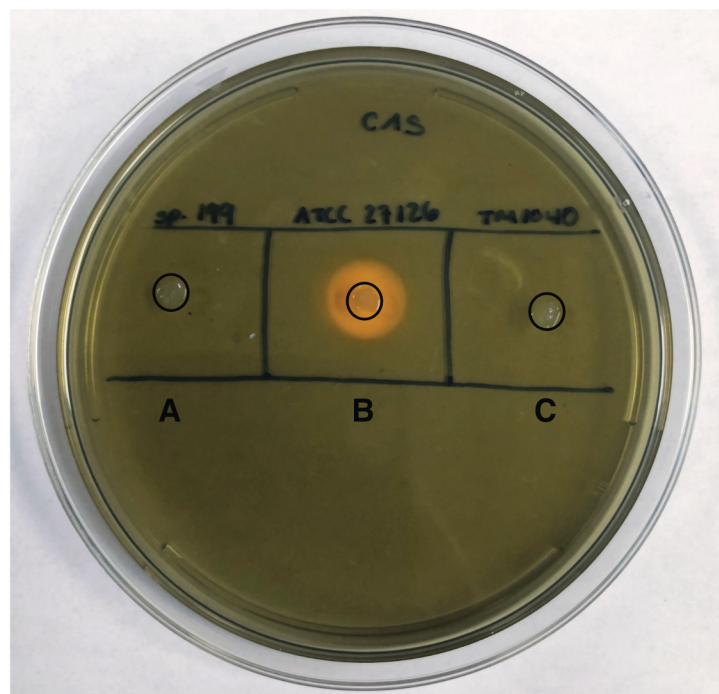

Supplement: FIG S3 [file mSystems.00070-20-sf003.pdf]

Figure S4

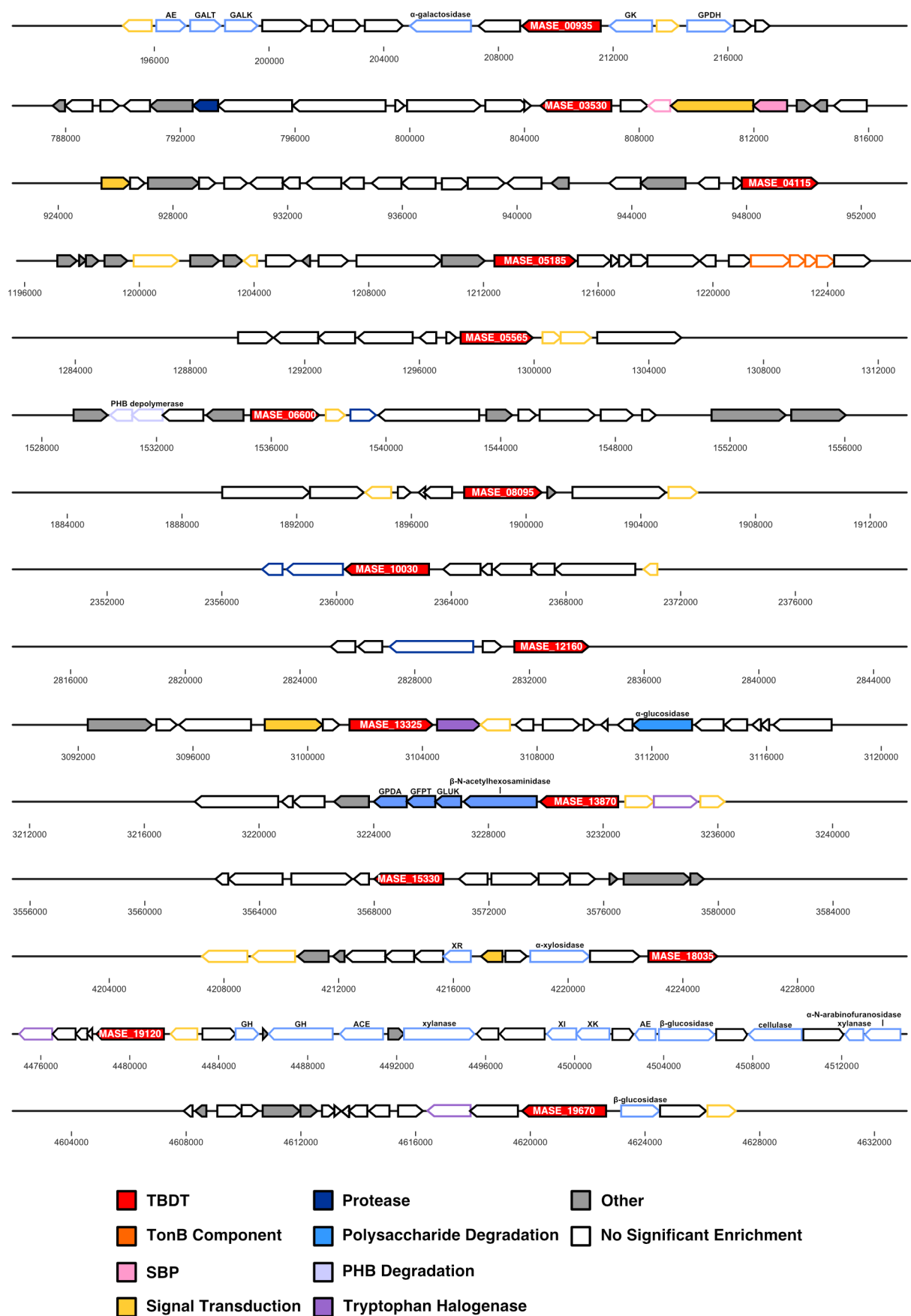

Supplement: FIG S4 [file mSystems.00070-20-sf004.pdf]

Figure S5

**Fold Change  $\geq 2$**

● Carbon limitation

● Iron limitation

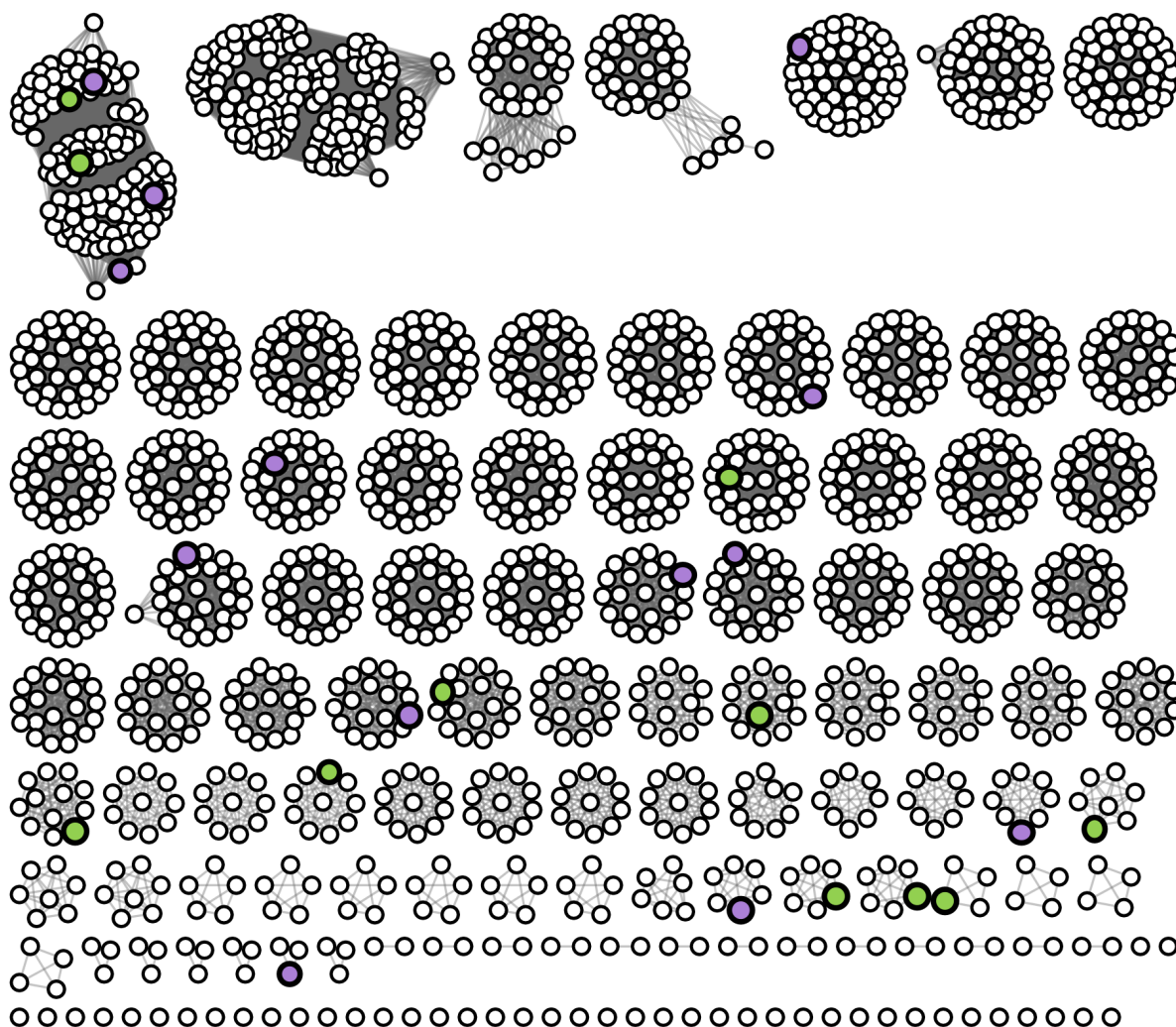

Supplement: FIG S5 [file mSystems.00070-20-sf005.pdf]

Figure S6

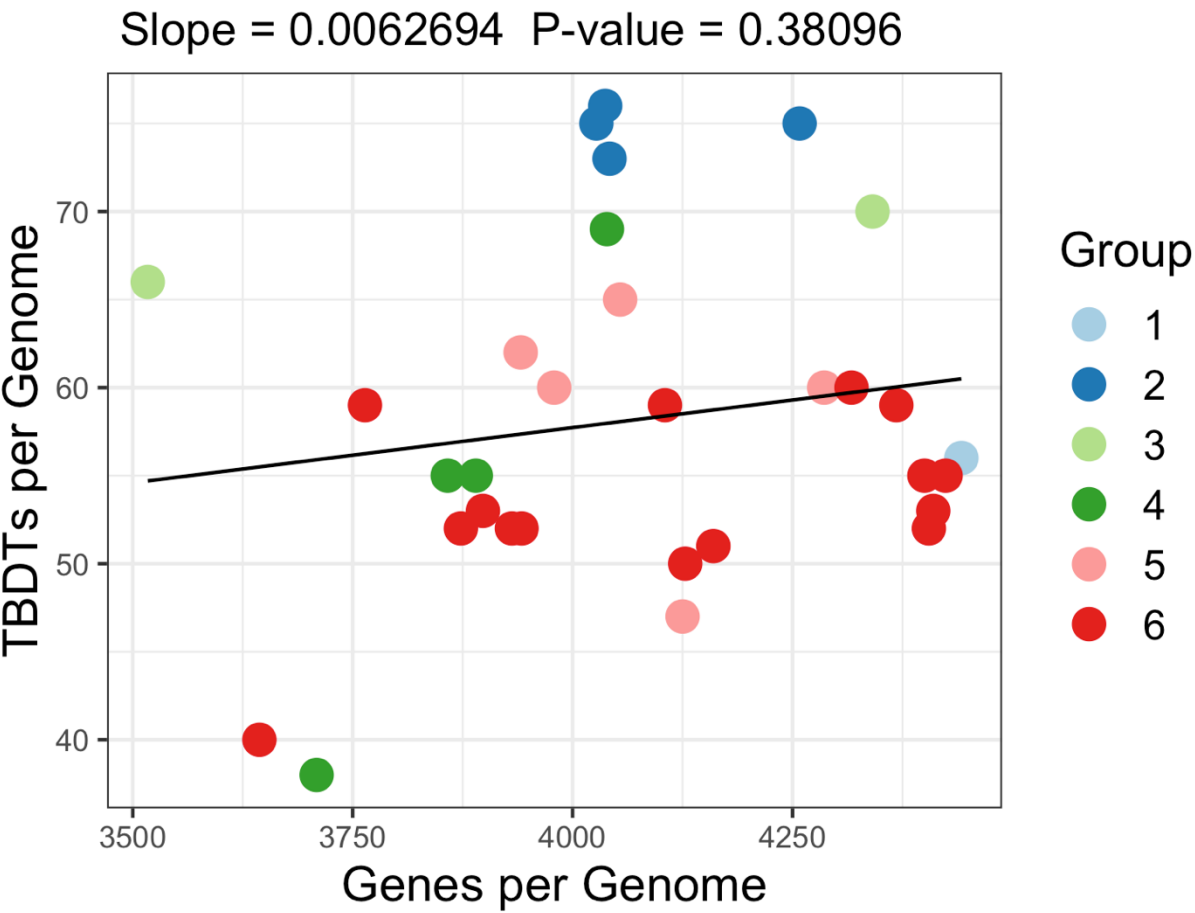

Supplement: FIG S6 [file mSystems.00070-20-sf006.pdf]
