# Supplementary material for: Transcriptomic Study of Substrate-Specific Transport Mechanisms for Iron and Carbon in the Marine Copiotroph Alteromonas macleodii
Source: mSystems. 2020 Apr 28;5(2):e00070-20. doi: 10.1128/mSystems.00070-20 (PMC7190382; doi:10.1128/mSystems.00070-20)
Supplement: TEXT S1 [file mSystems.00070-20-s0001.pdf]

Supplemental Text for

Transcriptomic study of substrate-specific transport mechanisms for iron and carbon in the marine copiotroph *Alteromonas macleodii*

Lauren E. Manck[#], Josh L. Espinoza, Christopher L. Dupont, and Katherine A. Barbeau

[#]Lauren E. Manck

lmanck@ucsd.edu

Material and Methods

Preparation of CAS Assay for Confirmation of Siderophore Production

This preparation of the chrome azurol S (CAS) assay for siderophore detection was adapted from Schwyn and Neilands (1). A base growth media was prepared in an acid-cleaned polycarbonate container by dissolving 0.885 g MOPS buffer in 37.5 mL of Milli-Q water and adjusting to pH 7. A sufficient amount of 0.2 µm filtered seawater was added to bring the total volume to 450 mL. 0.5 g of bacteriological peptone and 0.5 g of yeast extract were added to solution. The resulting solution was microwave sterilized and stirred overnight with 7% w/v Chelex 100 resin (Biorad) to remove contaminating trace metals. This base media solution was again filtered through a 0.2 µm filter to remove the Chelex resin and microwave sterilized an additional time.

In a separate acid-cleaned polycarbonate container, 0.03 g of CAS dye was dissolved in 25 mL Milli-Q water and 5 mL of a 1 x 10^{-3} M $FeCl_3$ solution (prepared in 1 x 10^{-2} M HCl) was added.

In a third acid-cleaned polycarbonate container, 0.0365 g of hexadecyltrimethlammonium bromide (HDTMA) was added to 20 mL Milli-Q water and sonicated for 20 minutes to achieve complete dissolution. Under stirring, the CAS-Fe solution was slowly added to the HDTMA solution yielding a dark blue solution. This solution was 0.2 μm filter-sterilized and stored at 4°C in the dark until use.

To prepare agar plates, 2% (w/v) agarose was added to the base growth media and melted in the microwave. The solution was allowed to cool to 55°C whereupon the CAS-Fe solution was added at an appropriate volume to comprise 10% of the final solution volume. A trace metal mix (see main text for details) was also added at this stage at a 1000-fold dilution. The resulting media was then poured into plates and allowed to solidify.

Single colonies of *A. macleodii* ATCC 27126 were spotted onto prepared plates along with strains lacking siderophore biosynthetic capabilities as negative controls. Within 24 hours distinctive orange halos around the ATCC 27126 colonies were detected, indicating siderophore production (Figure S3).

References

1. Schwyn B, Neilands JB. Universal chemical assay for the detection and determination of siderophores. Anal Biochem. 1987;160(1):47–56.
